# Unravelling associations between unassigned mass spectrometry peaks with frequent itemset mining techniques

**DOI:** 10.1186/s12953-014-0054-1

**Published:** 2014-11-18

**Authors:** Trung Nghia Vu, Aida Mrzic, Dirk Valkenborg, Evelyne Maes, Filip Lemière, Bart Goethals, Kris Laukens

**Affiliations:** Department of Mathematics and Computer Science, University of Antwerp, Antwerp, Belgium; Biomedical Informatics Research Center Antwerp (biomina), University of Antwerp / Antwerp University Hospital, Edegem, Belgium; Applied Bio & molecular Systems, VITO, Mol, Belgium; Center for Proteomics, Antwerp, Belgium; Interuniversity Institute for Biostatistics and Statistical Bioinformatics, Hasselt University, Hasselt, Belgium; KU Leuven, Functional Genomics and Proteomics lab, Leuven, Belgium; Department of Chemistry, University of Antwerp, Antwerp, Belgium

**Keywords:** Aberrant peaks, Unassigned masses, Pattern mining, Frequent itemset mining

## Abstract

**Background:**

Mass spectrometry-based proteomics experiments generate spectra that are rich in information. Often only a fraction of this information is used for peptide/protein identification, whereas a significant proportion of the peaks in a spectrum remain unexplained. In this paper we explore how a specific class of data mining techniques termed “frequent itemset mining” can be employed to discover patterns in the unassigned data, and how such patterns can help us interpret the origin of the unexpected/unexplained peaks.

**Results:**

First a model is proposed that describes the origin of the observed peaks in a mass spectrum. For this purpose we use the classical correlative database search algorithm. Peaks that support a positive identification of the spectrum are termed explained peaks. Next, frequent itemset mining techniques are introduced to infer which unexplained peaks are associated in a spectrum. The method is validated on two types of experimental proteomic data. First, peptide mass fingerprint data is analyzed to explain the unassigned peaks in a full scan mass spectrum. Interestingly, a large numbers of experimental spectra reveals several highly frequent unexplained masses, and pattern mining on these frequent masses demonstrates that subsets of these peaks frequently co-occur. Further evaluation shows that several of these co-occurring peaks indeed have a known common origin, and other patterns are promising hypothesis generators for further analysis. Second, the proposed methodology is validated on tandem mass spectrometral data using a public spectral library, where associations within the mass differences of unassigned peaks and peptide modifications are explored. The investigation of the found patterns illustrates that meaningful patterns can be discovered that can be explained by features of the employed technology and found modifications.

**Conclusions:**

This simple approach offers opportunities to monitor accumulating unexplained mass spectrometry data for emerging new patterns, with possible applications for the development of mass exclusion lists, for the refinement of quality control strategies and for a further interpretation of unexplained spectral peaks in mass spectrometry and tandem mass spectrometry.

**Electronic supplementary material:**

The online version of this article (doi:10.1186/s12953-014-0054-1) contains supplementary material, which is available to authorized users.

## Background

Mass spectrometry is the *de facto* standard technique used to identify proteins, by matching experimental spectral masses with calculated theoretical masses. Even high quality spectra that yield confident identifications often contain a significant number of unexplained masses. These masses are typically not further analyzed. At the level of MS^1^ survey scans, unassigned masses are usually assumed to be contaminants, post-translationally or chemically modified peptides, or non-protein components of samples. At the level of MS^2^ fragment spectra, unexplained masses may be caused by modifications at the level of individual amino acids, by contaminants or by atypical peptide fragmentation mechanisms.

Several authors [1,2] have investigated the nature of unexplained masses in experimental MS^1^ spectra, and described several common classes, including keratin peptides, common and less common autolysis peptides, matrix-alkali clusters, random cleavage products, chemical modification products and other unexplained yet commonly observed masses. Comparison between theoretical and experimental mass spectra of standard proteins showed that contamination accounts for most of the unmatched masses [3]. Furthermore it is clear that the commonly held rules [4] for the specificity of tryptic cleavage are an oversimplification, mainly because of effects of neighboring residues [5], experimental conditions, and contaminants present in the enzyme sample. The presence of such unexplained information undoubtedly affects downstream analysis and data interpretation. Software implementations use these unmatched masses to attempt to discover unexpected modifications [6,7] or to perform a second round search for complex proteins [8]. For example, FindPept [9] can identify unmatched masses resulting from unspecific cleavages in peptide fingerprint protein identifications. Tools for spectrum based protein identification eliminate masses from contaminants in a preprocessing step [10,11]. Trypsin autolysis and MALDI matrix clusters are often intense peaks in mass spectra of protein digests and can also be used for mass calibration and normalization [12,13]. All studies concerning the origin of unexplained aberrant masses took into account the occurrence of one mass at the time. However they did not yet investigate or use the fact that frequently co-occurring unassigned peaks are likely to have a common origin as they are associated to each other via the parent molecule.

At the level of MS^2^ peptide fragmentation spectra, several computational approaches are available to interpret spectra that do not yield a clear match in a conventional database searching strategy, ranging from error tolerant searching to de novo sequencing. None of these approaches can guarantee that all peaks within the spectrum are explained, even if a reliable identification is obtained for the spectrum as a whole [14]. New approaches to elucidate the relationships between unexplained fragment ion peaks are therefore welcome. [15].

In this work, we introduce a framework to discover frequently occurring aberrant fragments in mass spectrometry data. We propose a frequent itemset mining approach to reveal which peaks are associated, and are thus for example, likely to have a common origin. At MS^1^ level, the approach takes historical laboratory data to mine for interesting patterns that might originate from common contaminants, matrix akali clusters, etc. The methodology is further demostrated to be extendable, with specific adaptations, to MS^2^ peptide fragmentation data. For a given experimental spectrum, the system can reveal known patterns and recommend which peaks are potentially interesting for further analysis, similar to recommendation systems that are well known in other fields.

## Results and discussion

### Spectral model

**Figure 1:**
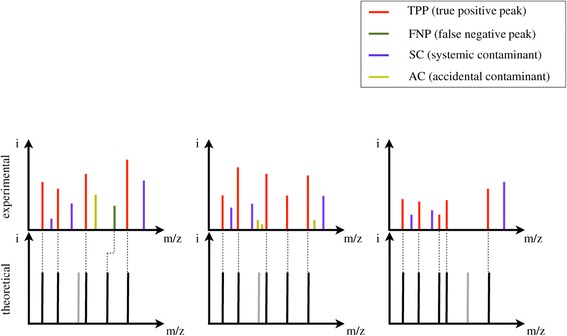
**Illustration of the origin of different peak classes in three experimental spectra (top), and their corresponding theoretical spectrum matches (bottom).** See text for an explanation of each of the four fragment classes.

We propose a data mining workflow to find interesting patterns based on the hypothesis of a spectral model. In this spectral model, illustrated in Figure 1, we distinguish observed ions according to their origins based on a correlative database search. A single MS^1^ spectrum from a tryptic digested protein then contains masses originating from four possible sources:

- TPP or true positive peaks, which includes masses of the single protein or mix of proteins that is to be identified in the peptide mass fingerprint. A typical search algorithm, such as Mascot [8], detects these masses and matches them to the theoretical spectrum generated from a protein sequence database.
- FNP or false negative peaks, which are the masses that correspond to the peptide, but that are not positively identified as such. For example, they may be the result of a modification of the peptide that is expected to be unmodified or due to semi-tryptic digests.
- SC or systematic contaminants, which are systematically generated by certain sources, for example human keratin, trypsin autolysis fragments, matrix cluster, tap tag fragments… One of their features is that they occur frequently over multiple spectra.
- AC or accidental contaminants, which correspond to accidental contaminants that are specific or unique to the spectrum under investigation and may change over time.

More formally, consider *S* = {*s*_1_,..*s*_*N*_}, a set of spectra in a dataset. Let *M*_*i*_ = {*m*_1_,..*m*_*n*_} be the set of masses of a spectrum *s*_*i*_ ∈ *S* . Let *T* = {*t*_1_,..*t*_*h*_} be the set of theoretical masses of the peptides that confidently match the spectrum. Thus, the model of spectrum *s*_*i*_ by the set of masses *M*_*i*_ is

$$ {M}_i=TPP\left({M}_i,T\right)\cup FNP\left({M}_i,T\right)\cup SC\left({M}_i,S\right)\cup AC\left({M}_i\right) $$

where each term on the right hand side of the equation is a subset of all theoretical masses, according to the following criteria:

- *TPP*(*M*_*i*_, *T*) ⊆ {*m*_*j*_|*m*_*j*_ = *t*_*v*_, *t*_*v*_ ∈ *T*}
- *FNP*(*M*_*i*_, *T*) ⊆ {*m*_*j*_|*m*_*j*_ = *t*_*v*_ + *m*_*mod*_, *t*_*v*_ ∈ *T*, *m*_*mod*_ is the mass of a peptide modification}
- *SC*(*M*_*i*_, *S*) ⊆ {*m*_*j*_|*m*_*j*_ ∈ *X*, *X* ⊆ *M*_*i*_, *support*(*X*, *S*) ≥ *σ*}(*)
- *AC*(*M*_*i*_, *S*) ⊆ *M*_*i*_\{*TPP*(*M*_*i*_, *T*) ∪ *FNP*(*M*_*i*_, *T*) ∪ *SC*(*M*_*i*_, *S*)}

(*): In more detail: A subset *X* = {*x*_1_,..*x*_*k*_} ⊆ *M*_*i*_ is an itemset of *M*_*i*_. Support of *X* is the number of spectra in the dataset containing *X*: *support*(*X*,*S*) = |{(*M*_*i*_*, s*_*i*_) |*s*_*i*_ ∈*S*, *X* ⊆ *M*_*i*_}|. *σ* is a minimal support threshold with 0 ≤ *σ* ≤ |*S*| that determines whether an itemset is frequent or not.

The method in this paper addresses the detection and interpretation of SC masses, without prior knowledge or assumptions regarding their sources. The first motivation for this goal is that a correct assignment of certain peaks of a spectrum to the SC class can facilitate and improve the correct assignment of all other masses in the spectra. Second, automatic and timely discovery of systematic contaminants, in particular if they are new and previously unseen, is an essential factor for computational quality assessment in mass spectrometry based proteomics, a field that is currently rapidly gaining momentum [16,17]. This detection of SC masses in unassigned spectral data is based on the fact that (1) by definition, systematic contaminants are characterized by their relatively high frequency of occurrence over multiple spectra and (2) each systematic contaminant may give rise to a set of masses that frequently co-occur.

This expected high frequency of peaks coming from systematic contaminants as well as the fact that ions originating from the same source tend to co-occur makes frequent itemset mining an attractive candidate technique for their detection. Frequent itemset mining is a class of pattern detection techniques that is specifically designed to discover co-occurring items in transactional datasets. The archetypical example of frequent itemset mining is the discovery of products that are frequently purchased together from mining large numbers of supermarket basket transactions. The study of such associations, for example the observation that beer and chips are frequently bought together, is a computationally non-trivial problem for which various algorithms have been developed, as reviewed before [18]. Despite the explosive number of possible patterns with growing datasets, frequent itemset mining techniques are available to efficiently extract all possible patterns even from large and complex databases.

### Pattern mining reveals associations between systematic contaminant peaks

The employed frequent itemset mining strategy looks for patterns that consist of peaks that frequently co-occur in the dataset. While many of such patterns are usually present, often with significant overlap, we have opted for a methodology that does not attempt to exhaustively list all patterns that are present but rather presents only the most informative patterns relevant for the dataset. The *mtv* algorithm [19] specifically attempts to extract the few most representative patterns from the data, i.e. that “summarize” the data in the most efficient way. It uses a probabilistic maximum entropy model to iteratively find the most interesting patterns. This approach guarentees that the found patterns are descriptive and non-redundant.

**Figure 2:**
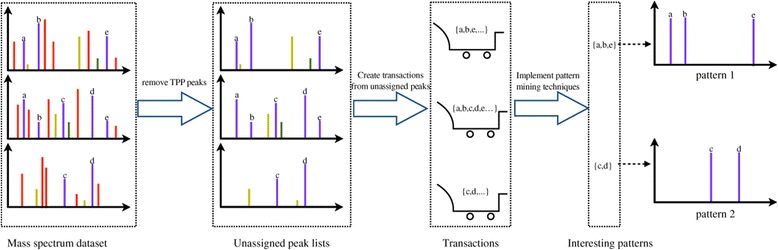
**Framework of a data mining approach to detect systematic contaminants at the MS** ^**1**^ **level.**

The framework of the approach at the MS^1^ level is presented in Figure 2. In this experiment we employ the term “transaction” to define a set of unassigned nominal masses extracted from the same spectrum. These transactions form the basis for the itemset mining workflow.

**Figure 3:**
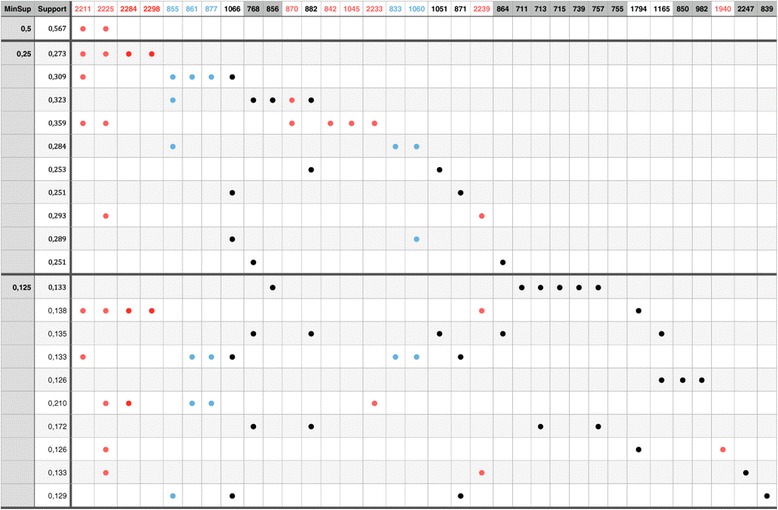
**Patterns retrieved from mining the top 10 frequent itemsets (using the** ***mtv*** **algorithm) from unexplained peaks in a peptide mass fingerprint datasets.** Each row corresponds to a pattern. Masses present in the pattern are indicated as dots, with a color representing their origins (red = trypsin, blue = matrix cluster, black = unknown). Results for three different support thresholds (0.5, 0.25 and 0.125) are shown.

Application of the approach using varying support (the fraction of the transactions containing the pattern) thresholds yields a series of patterns, represented in Figure 3. Each pattern consists of an “informative” set of peaks that co-occur more frequently than expected from the individual frequencies of the items under the assumption of independence. In this figure each row corresponds to a pattern, and the masses that it contains are represented with a dot. For several masses we already know the origin, based on background laboratory knowledge and past publications describing contaminants. For known contaminants, the dots (and corresponding columns) are colored according to their origin.

For a support threshold of 0.5, i.e. all considering items occur in at least 50% of the transactions, only one pattern is found that contains more than one mass. Its two masses (2211 and 2225) co-occur in more than half of the spectra (support =0.567) and they share a common, trivial origin: trypsin. Decreasing the minimal support to 0.25, leads to 10 new patterns that contain more unexplained masses and are less omnipresent, i.e. at least 25%. The two most obvious patterns are both an extension of the tryptic pattern shown before with respectively two and 4 additional masses, which all have a known tryptic origin. From the 10 patterns, there are two that contain peaks with more than one known origin, or more specifically two origins. Both contain only one peak from the second origin. Other patterns are entirely homogeneous regarding their origin (4 patterns), consisting of a mix of peaks with known and unknown origins (1 pattern), and entirely of unknown origin (3 patterns). A further decrease of the support threshold to 0.125, reveals, in the top 10 patterns, 2 patterns of heterogeneous origins, while all the other patterns consist of unknown peaks (4/10) or a mix of known and unknown patterns (4/10). Decreasing the support threshold leads to longer patterns (see Additional file 1: Figure S1). For example, with a threshold of 0.031, a new pattern emerges that contains 20 novel associated but unexplained peaks. Despite their size and high frequency, such patterns would typically remain undetected and thus unknown when a conventional proteomics data processing is followed. The observation of such a pattern obviously calls for further inspection and the elucidation of its origins can be of great help for the improvement of experimental workflows.

### Significance of the revealed patterns

In order to improve confidence in our finding, we evaluate the statistical properties of the method by a simulation study. Therefore, we investigate whether items with the same known origin are more often associated in a single pattern by comparing the homogeneity of observed patterns with the homogeneity expected by chance. The latter distribution is estimated by randomly shuffling the known labels. Only items in the set of patterns found by *mtv* with a given support value were considered. Firstly, the number of non-homogeneous patterns of the set is calculated. Next, the labels of the items are shuffled 1000 times to create 1000 new random pattern sets. The number of non-homogeneous patterns is also computed for each new set to create an empirical null distribution. Subsequently, the empirical cumulative distribution function is applied to the distribution and the number of non-homogeneous patterns of the original set. Finally, the percentile value is generated. The results are summarized in Table 1. This table demonstrates that the patterns are more homogeneous than if they would be random, and confirms that meaningful associations are generated through the frequent itemset mining approach. The method also generates various associations between unknown peaks that are interesting for further analysis. Similar results were obtained when the method was applied to an entirely independent dataset, generated with a different instrument in a different laboratory (results not shown).

**Table 1:** **Summary of frequent itemset mining results for varying minimum support thresholds**

**Support**	**Mean**	**Median**	**# non-homogeneous patterns**	**Empirical cumulative distribution**
**0.5**	Na	Na	Na	Na
**0.25**	4.993	5	2	0.02
**0.125**	4.459	4	2	0.068
**0.0625**	4.839	5	3	0.163
**0.03125**	3.987	4	3	0.341
**0.015625**	4.163	4	3	0.294
**0.007812**	3.233	3	2	0.218

### Expanding the approach to the MS^2^ level

**Figure 4:**
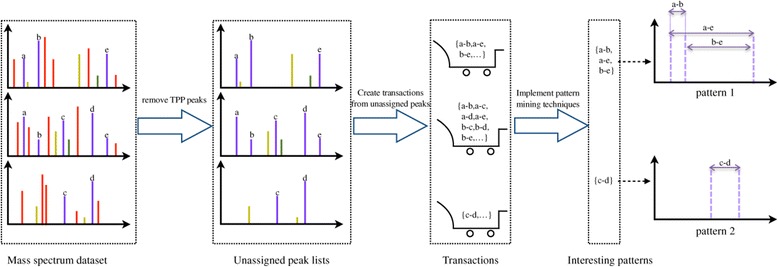
**Framework of a data mining approach to detect systematic contaminants at the MS** ^**2**^ **level.**

In our second analysis, we applied the approach to a publicly available MS^2^ dataset. In this experiment we explored the relation between unannotated peaks and a modification of the peptide. In contrast to MS^1^, rather than the absolute masses, the mass differences between peaks within a fragmentation spectrum are the most descriptive features that are relevant for structural elucidation. We therefore adapted the workflow to use the mass differences between unexplained peaks. In addition, known and annotated amino acid modifications present within the spectrum are encoded as additional items. The framework of the approach at the MS^2^ level is presented in Figure 4. This workflow resulted in 7,085 transactions were created from the human plasma library.

The relation between unannotated peaks and the modifications of the peptides was subsequently investigated. The peptide modifications were encoded as the items and included into transactions. For this purpose, a modification at a specific site of a peptide sequence is represented by an integer value, *ModVal*, as defined in formula (1).

1 $$ ModVal = 1000 + mas{s}_{container}+mas{s}_{modification} $$

where; *mass*_*container*_ is the mass of amino acid that the modification is attached to; *mass*_*modification*_ is the mass of the modification. The addition of 1,000 is to separate the mass item from any item describing the ion mass differences. The *mass*_*container*_ that terminates the peptide sequence is 1.0. *ModVal* =1000 if the peptide does not contain any modification. For example, a phosphorylation modification at amino acid S of a peptide sequence is encoded by *ModVal* =1000 + 87 + 80 or *ModVal* = 1167, where 87 is the mass of S and 80 is the mass of the phosphorylation modification. Since the modification masses and amino acid masses are known, it is straightforward to check that the values of Modval are unique for each peptide modification. Thus, we encoded the peptide modifications as the items and put the encoded items into transactions. After obtaining all final transactions, frequent itemset techniques were applied to extract interesting patterns.

Interesting patterns discovered by *mtv* for a support threshold of 0.005 are shown in Table 2. The first pattern contains the modification codes 1230 and 1357 which are from the same modification TMT6plex at N terminate and amino acid K sites, respectively. Thus, it is clear that items 100, 101, 102, 103 and 104 usually occur with TMT6plex in more than 5% of transactions. We further checked this by taking a look at spectral mass differences. Mass differences 100, 101, 102, 103 and 104 result from the subtraction of the TMT6plex peak at N terminate (230) and the reporter ions from the distribution of TMT6plex: 130, 129, 128, 127 and 126, respectively. Therefore, the pattern suggests that the corresponding peaks should be annotated and properly re-weighed in the spectrum similarity scoring of library searching software. This trivial example with a known modification demonstrates that itemset mining can also be used to find meaningful patterns in unexplained MS^2^ data.

**Table 2:** **Patterns retrieved from mining the top 10 frequent itemsets (using the** ***mtv*** **algorithm) from unexplained peaks in the Human Plasma dataset**

**Support**	**Itemsets**
**0.0542**	**100 101 102 103 104 1230 1357**
0.0134	21 22 23
0.0110	25 26 27
0.0064	27 28 29 30
0.0096	30 31 32
0.0097	112 113 114
0.0086	86 86 88
0.0061	22 24 27 33
0.0058	23 24 34 35
0.0082	48 49 50

## Conclusions

To conclude, this study presents an application of frequent itemset mining to reveal patterns in unlabeled mass spectrometry data. A model of a spectrum containing true positive mass of protein (TPP), false negative peaks (FNP), systematic contaminants (SC) and accidental contaminants (AC) was proposed. We showed that frequent itemset mining techniques can be used to uncover potential systemic contaminants and generate new hypotheses regarding the source of unexplained data. An experimental evaluation at the MS^1^ level showed that patterns found are largely consistent with known origins, while interesting new patterns that call for further investigation were also found. The approach is very simple to apply, and works with default values. Variation with the thresholds of minimum support allows exploring different mining depths. While the method is specifically evaluated using peptide mass fingerprinting data, it is potentially interesting as a hypothesis generator for any dataset in which associations between unexplained peaks are relevant. The method can be incorporated to generate interesting patterns that emerged throughout history in historical laboratory data to. For new experimental data, such patterns can give valuable hints for interpretation, quality control and data dependent acquisition workflows. At the MS^2^ level, we mined interesting patterns on human plasma public spectral library. One interesting pattern was found and could be clearly explained via the features of the known modification. Thus, all results demonstrated the strengths of pattern mining techniques and revealed their promising application in mass spectometry data analysis. While in this paper we have opted for an algorithm that produces a small and non-redundant list of the most descriptive patterns, the workflow can be easily adapted to generate larger lists of patterns, if desired, by replacing the *mtv* algorithm with a more exhaustive pattern mining algorithm.

## Methods

For the experimental evaluation at the MS^1^ level we used a dataset [20] consisting of 443 MALDI TOF spectra from proteins extracted from peripheral blood mononuclear cells (PBMCs) that are isolated from human blood using Leucosep tubes (density centrifugation), separated with gel electophoresis (2D-DIGE) in-gel, digested with trypsin, and analyzed with matrix assisted laser desorption/ionization time-of-flight mass spectrometry (MALDI-TOF MS) (Ultraflex II, Bruker). More information regarding the dataset and parameters is described in Maes et al [20].

The workflow at the MS^1^ level is presented in Figure 2. After preprocessing the spectral data, the resulting peak lists were compared to the relevant SwissProt [21] sequence databases using a Mascot server [8] (version 2.2.0). Subsequently, all assigned peaks from spectra that yielded positive hits in Mascot (95% confidence) were eliminated. From the remaining spectra, all unassigned peaks were extracted, and discretized to their nominal masses. The *mtv* algorithm was used with its default parameters.

The human plasma public library (built in 08-2012) that can be downloaded from http://www.peptideatlas.org/builds/ was used for experimental evaluation at the MS^2^ level. The consensus spectrum of an individual peptide in the library contains peaks appearing over 60% of the peptide’s replicates, which means that peaks are relatively consistent. The workflow at MS^2^ level is presented in Figure 4. Again a correlative database search is performed to annotate peaks in the tandem mass spectrum. The unannotated peaks of a peptide were extracted if their intensities are significant (greater than 20% of top peak of spectrum). Subsequently, their nominal mass differences were used to create a data transaction. We retained only mass differences in the range from 20 to 600. The upper bound corresponds to the maximum mass addition observed with ICAT labeling, where labeled Cysteine could yield to 554 Da extra. The lower bound removes noise or common simple neutral losses (water, ammonia, proton, etc.).

## Additional file

Additional file 1: Figure S1. Patterns retrieved from mining the top 10 frequent itemsets (using the *mtv* algorithm) from unexplained peaks in a peptide mass fingerprint datasets. Results for all different support thresholds are shown. Each line corresponds to a pattern. Masses present in the pattern are indicated as dots, with a color representing their origins (red = trypsin, blue = matrix cluster, green = keratin, black = unknown).

## Abbreviations

PMF: Mass finger printing
MALDI: Matrix assisted laser desorption/ionization
TPP: True Positive Peaks
FNP: False Negative Peaks
SC: Systematic contaminants
AC: Accidental contaminants
MS: Mass Spectrometry
MS^2^: Tandem Mass Spectrometry

## References

[1] Parker KC, Garrels JI, Hines W, Butler EM, McKee AH, Patterson D, Martin S (1998). Identification of yeast proteins from two-dimensional gels: working out spot cross-contamination. Electrophoresis.

[2] Karty JA, Ireland MME, Brun YV, Reilly JP (2002). Artifacts and unassigned masses encountered in peptide mass mapping. J Chromatogr B.

[3] Ding Q, Xiao L, Xiong S, Jia Y, Que H, Guo Y, Liu S (2003). Unmatched masses in peptide mass fingerprints caused by cross-contamination: an updated statistical result. Proteomics.

[4] Keil B (1992). Specificity of Proteolysis.

[5] Fannes T, Vandermarliere E, Schietgat L, Degroeve S, Martens L, Ramon J (2013). Predicting tryptic cleavage from proteomics data using decision tree ensembles. J Proteome Res.

[6] Barsnes H, Mikalsen S-O, Eidhammer I (2006). MassSorter: a tool for administrating and analyzing data from mass spectrometry experiments on proteins with known amino acid sequences. BMC Bioinformatics.

[7] Barsnes H, Eidhammer I, Cruciani V, Mikalsen S-O (2008). Protease-dependent fractional mass and peptide properties. Eur J Mass Spectrom (Chichester, Eng).

[8] Perkins DN, Pappin DJC, Creasy DM, Cottrell JS (1999). Probability-based protein identification by searching sequence databases using mass spectrometry data. Electrophoresis.

[9] Gattiker A, Bienvenut WV, Bairoch A, Gasteiger E (2002). FindPept, a tool to identify unmatched masses in peptide mass fingerprinting protein identification. Proteomics.

[10] Schmidt F, Schmid M, Jungblut PR, Mattow J, Facius A, Pleissner KP (2003). Iterative data analysis is the key for exhaustive analysis of peptide mass fingerprints from proteins separated by two-dimensional electrophoresis. J Am Soc Mass Spectrom.

[11] Tiengo A, Barbarini N, Troiani S, Rusconi L, Magni P (2009). A Perl procedure for protein identification by Peptide Mass Fingerprinting. BMC Bioinformatics.

[12] Harris WA, Janecki DJ, Reilly JP (2002). Use of matrix clusters and trypsin autolysis fragments as mass calibrants in matrix-assisted laser desorption/ionization time-of-flight mass spectrometry. Rapid Commun Mass Spectrom.

[13] Fonville JM, Carter C, Cloarec O, Nicholson JK, Lindon JC, Bunch J, Holmes E (2012). Robust data processing and normalization strategy for MALDI mass spectrometric imaging. Anal Chem.

[14] Tabb DL, Friedman DB, Ham A-JL (2006). Verification of automated peptide identifications from proteomic tandem mass spectra. Nat Protoc.

[15] Han X, He L, Xin L, Shan B, Ma B (2011). PeaksPTM: mass spectrometry-based identification of peptides with unspecified modifications. J Proteome Res.

[16] Martens L (2010). A report on the ESF workshop on quality control in proteomics. Mol BioSyst.

[17] Gu Q, Yu L-R (2014). Proteomics quality and standard: from a regulatory perspective. J Proteome.

[18] Naulaerts S, Meysman P, Bittremieux W, Vu TN, Vanden Berghe W, Goethals B, Laukens K (2013). A primer to frequent itemset mining for bioinformatics. Brief Bioinform.

[19] Mampaey M, Tatti N, Vreeken J (2011). Tell me what i need to know: succinctly summarizing data with itemsets. Proceedings of the 17th ACM SIGKDD International Conference on Knowledge Discovery and Data Mining.

[20] Maes E, Landuyt B, Mertens I, Schoofs L (2013). Interindividual variation in the proteome of human peripheral blood mononuclear cells. PLoS One.

[21] Bairoch A, Boeckmann B (1994). The SWISS-PROT protein sequence data bank: current status. Nucleic Acids Res.

