# Supplementary material for: Unravelling associations between unassigned mass spectrometry peaks with frequent itemset mining techniques
Source: Proteome Sci. 2014 Nov 18;12:54. doi: 10.1186/s12953-014-0054-1 (PMC4243190; doi:10.1186/s12953-014-0054-1)
Supplement: Additional file 1: Figure S1. — Patterns retrieved from mining the top 10 frequent itemsets (using the mtv algorithm) from unexplained peaks in a peptide mass fingerprint datasets. Results for all different support thresholds are shown. Each line corresponds to a pattern. Masses present in the pattern are indicated as dots, with a color representing their origins (red = trypsin, blue = matrix cluster, green = keratin, black = unknown). [file 12953_2014_54_MOESM1_ESM.docx]

Figure S1. Patterns retrieved from mining the top 10 frequent itemsets (using the mtv algorithm) from unexplained peaks in a peptide mass fingerprint datasets. Each line corresponds to a pattern. Masses present in the pattern are indicated as dots, with a color representing their origins (red=trypsin, blue=matrix cluster, green=keratin, black=unknown). Results for all different support thresholds are shown.
